# Case Report: Hereditary Fibrosing Poikiloderma With Tendon Contractures, Myopathy, and Pulmonary Fibrosis (POIKTMP) Presenting With Liver Cirrhosis and Steroid-Responsive Interstitial Pneumonia

**DOI:** 10.3389/fgene.2022.870192

**Published:** 2022-05-05

**Authors:** Michiko Takimoto-Sato, Toshinari Miyauchi, Masaru Suzuki, Hideyuki Ujiie, Toshifumi Nomura, Tomoo Ikari, Tomohiko Nakamura, Kei Takahashi, Machiko Matsumoto-Sasaki, Hirokazu Kimura, Hiroki Kimura, Yuichiro Matsui, Takashi Kitagataya, Ren Yamada, Kazuharu Suzuki, Akihisa Nakamura, Masato Nakai, Takuya Sho, Koji Ogawa, Naoya Sakamoto, Naoko Yamaguchi, Noriyuki Otsuka, Utano Tomaru, Satoshi Konno

**Affiliations:** ^1^ Department of Respiratory Medicine, Faculty of Medicine and Graduate School of Medicine, Hokkaido University, Sapporo, Japan; ^2^ Department of Dermatology, Faculty of Medicine and Graduate School of Medicine, Hokkaido University, Sapporo, Japan; ^3^ Department of Dermatology, Faculty of Medicine, University of Tsukuba, Tsukuba, Japan; ^4^ Department of Orthopaedic Surgery, Faculty of Medicine and Graduate School of Medicine, Hokkaido University, Sapporo, Japan; ^5^ Department of Gastroenterology and Hepatology, Faculty of Medicine and Graduate School of Medicine, Hokkaido University, Sapporo, Japan; ^6^ Department of Pathology, Faculty of Medicine and Graduate School of Medicine, Hokkaido University, Sapporo, Japan

**Keywords:** POIKTMP, FAM111B, interstitial pneumonia, liver cirrhosis, case report

## Abstract

**Background:** Hereditary fibrosing poikiloderma with tendon contractures, myopathy, and pulmonary fibrosis (POIKTMP) is an extremely rare disease caused by mutations in FAM111B, and only approximately 30 cases have been reported worldwide. Some patients develop interstitial pneumonia, which may lead to progressive pulmonary fibrosis and poor prognosis. However, no effective treatment for interstitial pneumonia associated with POIKTMP has been reported. Here, we report an autopsy case of POIKTMP, wherein interstitial pneumonia was improved by corticosteroids.

**Case Presentation:** A 44-year-old Japanese man was referred to our hospital due to poikiloderma, hypotrichosis, and interstitial pneumonia. He developed progressive poikiloderma and muscle weakness since infancy. He also had tendon contractures, short stature, liver cirrhosis, and interstitial pneumonia. Mutation analysis of FAM111B revealed a novel and *de novo* heterozygous missense mutation, c.1886T > G (p(Phe629Cys)), through which we were able to diagnose the patient with POIKTMP. 3 years after the POIKTMP diagnosis, interstitial pneumonia had worsened. After 2 weeks of administrating 40 mg/day of prednisolone, his symptoms and lung shadows improved. However, he subsequently developed severe hepatic encephalopathy and eventually died of respiratory failure due to bacterial pneumonia and pulmonary edema. Autopsy revealed an unclassifiable pattern of interstitial pneumonia, as well as the presence of fibrosis and fatty degeneration in several organs, including the liver, kidney, skeletal muscle, heart, pancreas, and thyroid.

**Conclusions:** We report a case of POIKTMP in which interstitial pneumonia was improved by corticosteroids, suggesting that corticosteroids could be an option for the treatment of interstitial pneumonia associated with this disease.

## Introduction

Hereditary fibrosing poikiloderma with tendon contractures, myopathy, and pulmonary fibrosis (POIKTMP) is a recently described genetic disorder, caused by mutation in FAM111B, which is inherited in an autosomal dominant manner (Mercier et al., 2013). POIKTMP is an extremely rare disease, and only approximately 30 cases have been reported worldwide. Patients with POIKTMP develop poikiloderma during early infancy, predominantly in sun-exposed areas, as well as progressive muscle atrophy, tendon contractures, and sparse hair. Elevation of liver enzymes, cataracts, and exocrine pancreatic insufficiency were also observed. Some patients develop interstitial pneumonia, which may lead to progressive pulmonary fibrosis and poor prognosis due to respiratory failure in their 30–50 s (Khumalo et al., 2006; Mercier et al., 2015; Sanchis-Borja et al., 2018). To date, no effective treatment has been reported for this disease, including interstitial pneumonia.

Here, we report an autopsy case of POIKTMP in which interstitial pneumonia was improved by corticosteroids.

### Case Description

A 44-year-old Japanese man was referred to our hospital with various systemic symptoms that had gradually developed since infancy. His parents were non-consanguineous, and there was no family history of similar symptoms. He had a medical history of dysphagia and dysarthria from around the age of 35 years, and hearing loss occurred at approximately 40 years of age.

**FIGURE 1:**
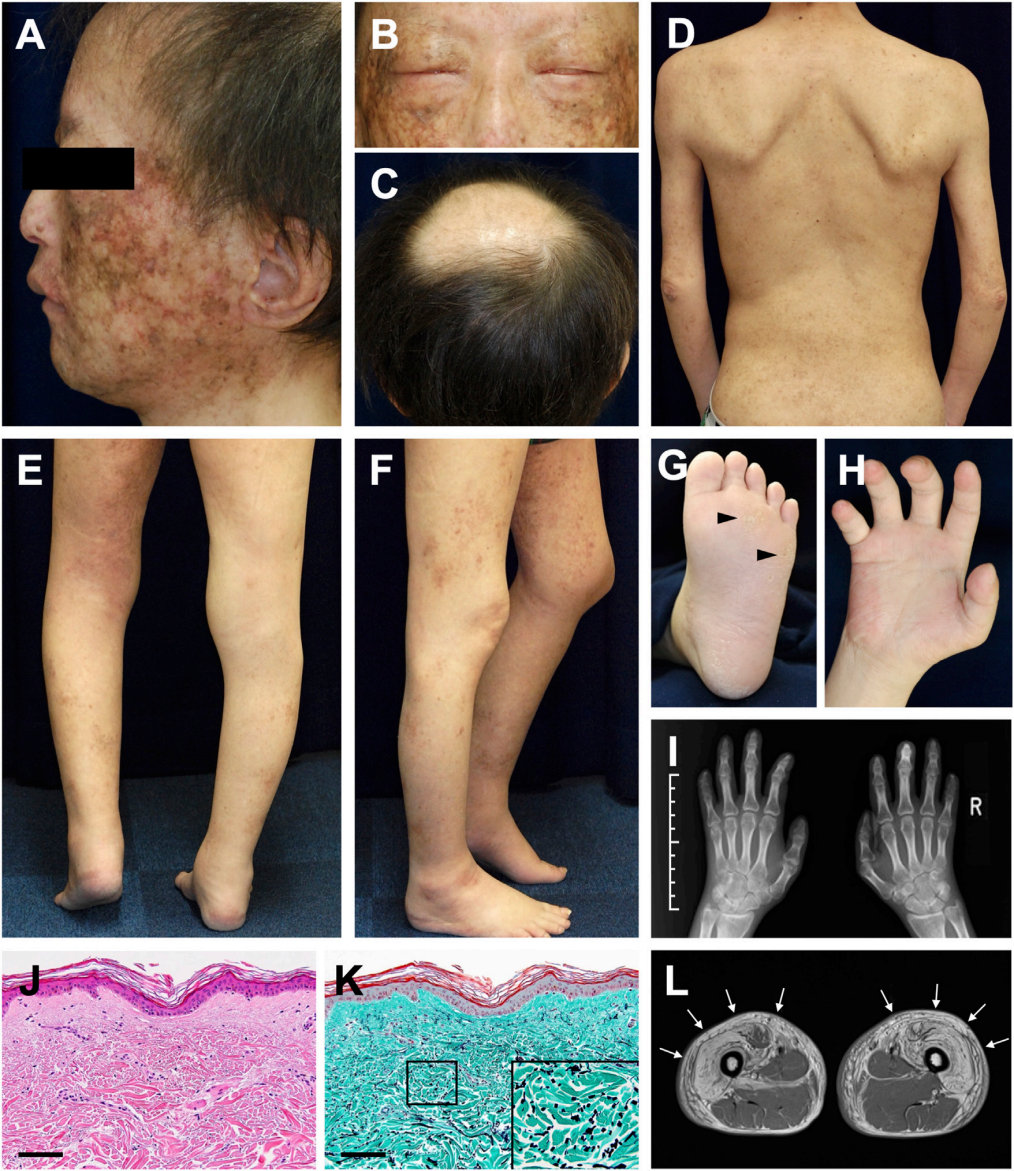
Clinical and histopathological manifestations **(A)** Poikiloderma with mottled pigmentation and telangiectasia on the face **(B)** Sparse eyebrows and eyelashes and lagophthalmos **(C)** Hairless on top of the head **(D)** Dry skin, mild eczema, and small pigmented macules with skin sclerosis **(E,F)** Mild lymphedema of the lower limbs **(E)** and contracture of the left Achilles tendon **(F) (G)** Mild hyperkeratosis with calluses (arrowheads) on the sole **(H)** Impaired extension of the fingers **(I)** Mild hypoplasia of the hands evaluated using X-ray photography **(J)** Pathological features of the non-sun-exposed area of the anterior chest. Hematoxylin and eosin (H&E) stain (scale bar, 100 μm) **(K)** Elastica–Masson staining. The inset shows a high-magnification image of the area indicated in the box (scale bar, 100 μm). **(L)** Diffuse bright appearance (white arrows) of the bilateral quadriceps muscles detected with muscle MRI (T1-weighted sequence).

Dermatological and orthopedic examinations revealed several characteristic manifestations. The patient had a short stature (149 cm) and presented with muscle weakness in all extremities. Poikiloderma with mottled pigmentation and telangiectasia was remarkable on the face (Figure 1A). The eyebrows and eyelashes were almost absent, and lagophthalmos due to skin sclerosis was observed (Figure 1B). Scalp hairs were also sparse, and the top of the head had been almost hairless since childhood (Figure 1C). The trunk and extremities showed dry skin, mild eczema, small pigmented macules, and skin sclerosis (Figure 1D). He had mild lymphedema of the lower limbs and a medical history of cellulitis due to this symptom. His left heel could not be placed on the ground due to Achilles tendon contracture, resulting in a constant flexed position of his left knee (Figures 1E,F). Mild hypoplasia of the hands and feet, multiple calluses on the soles, and impaired extension of the fingers were noted (Figures 1G–I). Histology of the non-sun-exposed skin showed epidermal atrophy and mild dermal fibrosis (Figure 1J). Fragmented elastic fibers were clearly observed with Elastica–Masson staining (Figure 1K). Muscle magnetic resonance imaging (MRI) findings showed severe fatty infiltration in the bilateral quadriceps muscles (Figure 1L), erector spinae muscles, and muscles in the upper extremities.

Further examinations revealed interstitial pneumonia, liver cirrhosis, and chronic kidney disease. A chest computed tomography (CT) scan revealed interstitial lung shadows, predominantly in the upper lobes, indicating the cause of his shortness of breath. His liver cirrhosis was graded as Child–Pugh class B and was accompanied by portal hypertension and esophageal varix. In this case, liver cirrhosis was diagnosed based on the findings of CT and ultrasonography, since a biopsy could not be performed. Ophthalmological examination revealed the presence of retinal rod dysfunction, lacrimal passage obstruction, and ptosis, but not cataract. Otolaryngological examination revealed atrophied vocal cords and mild sensorineural hearing loss.

### Diagnostic Assessment

**FIGURE 2:**
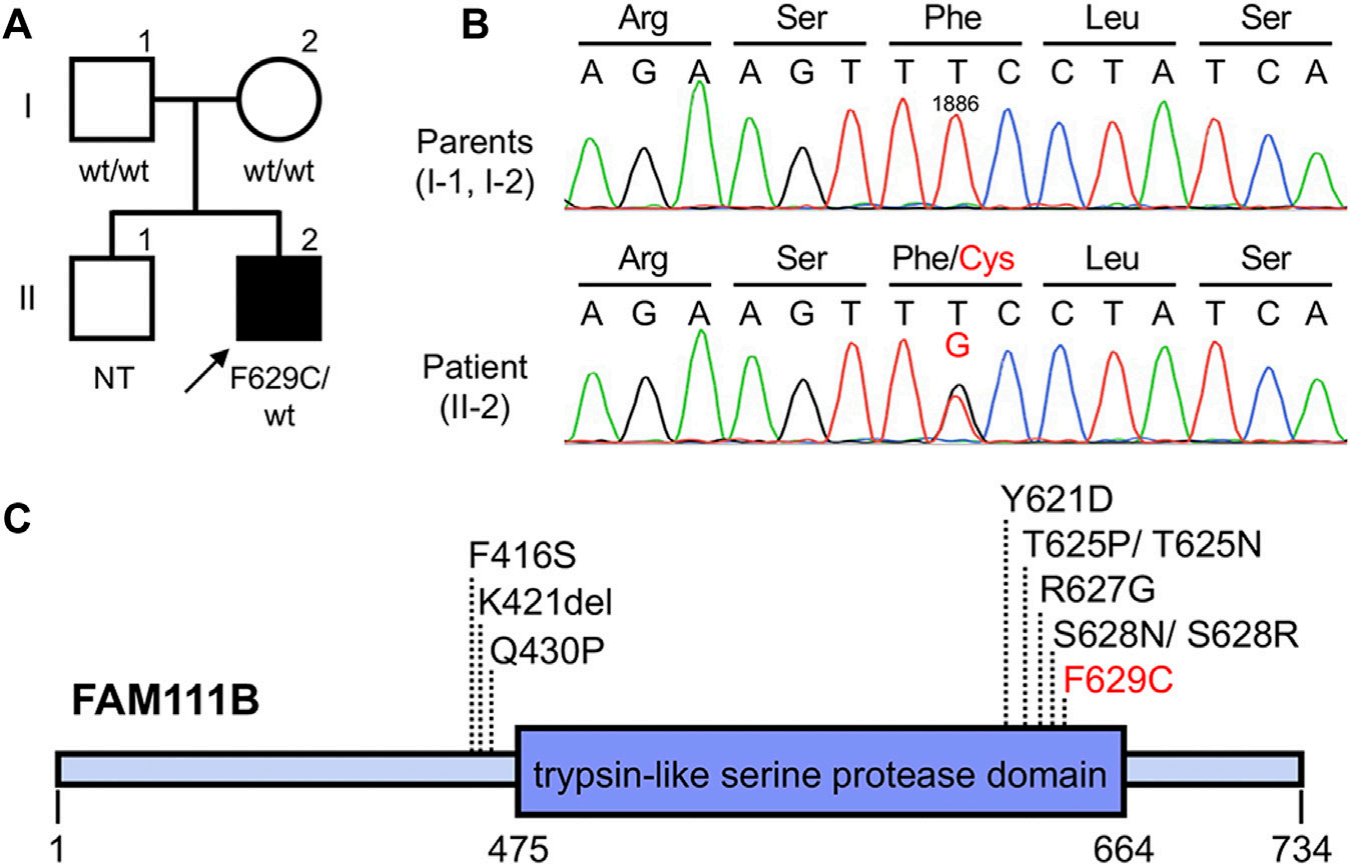
Pedigree and genetic analysis of FAM111B **(A,B)** Pedigree of this case **(A)**. The patient was heterozygous for **(C)**1886T > G (p(Phe629Cys)) in FAM111B, whereas his parents did not harbor this mutation **(B)**. wt, wild-type; NT, not tested **(C)** Overview of POIKTMP-related mutations detected in the present case and previous cases.

Based on the clinical findings described above, a diagnosis of POIKTMP was highly suspected. To confirm this, mutation analysis of FAM111B was performed. The patient and his parents provided written informed consent to participate in this study, in compliance with the Declaration of Helsinki. The Institutional Review Board at Hokkaido University Graduate School of Medicine approved this study (project no. 14–063). In brief, genomic DNA was extracted from peripheral blood using the QIAamp DNA Blood Maxi Kit (Qiagen, Hilden, Germany), and all exons and exon–intron boundaries of FAM111B were amplified using polymerase chain reaction (PCR). The PCR products were sequenced using an ABI 3130xl Genetic Analyzer (Applied Biosystems, Foster City, CA, United States), which led to the identification of a novel and *de novo* heterozygous missense mutation c.1886T > G (p(Phe629Cys)) (RefSeq accession number NM_198,947.4) in the patient (Figures 2A,B). This mutation was absent in 96 unrelated Japanese controls and in databases, such as the 1000 Genomes Browser, the Single Nucleotide Polymorphism Database, the Human Genetic Variation Database, and gnomAD. Notably, the position of the mutation was very close to that of other pathogenic mutations previously reported (Figure 2C), and the amino acid residue affected by this missense mutation was conserved among mammals (Mercier et al., 2015), suggesting the importance of the residue in the function of FAM111B. The pathogenicity was also estimated using *in silico* algorithms; PolyPhen2 (probably damaging), PROVEAN (deleterious), SIFT (damaging), and CADD score (25.4). Furthermore, it is classified as pathogenic also according to the ACMG guideline. Conversely, although RECQL4, the causative gene of Rothmund–Thomson syndrome (RTS) (which is the most common differential diagnosis), was also analyzed, no pathogenic mutations were detected in the patient (data not shown). These findings verified the diagnosis of POIKTMP, making this the first case in Japan.

**FIGURE 3:**
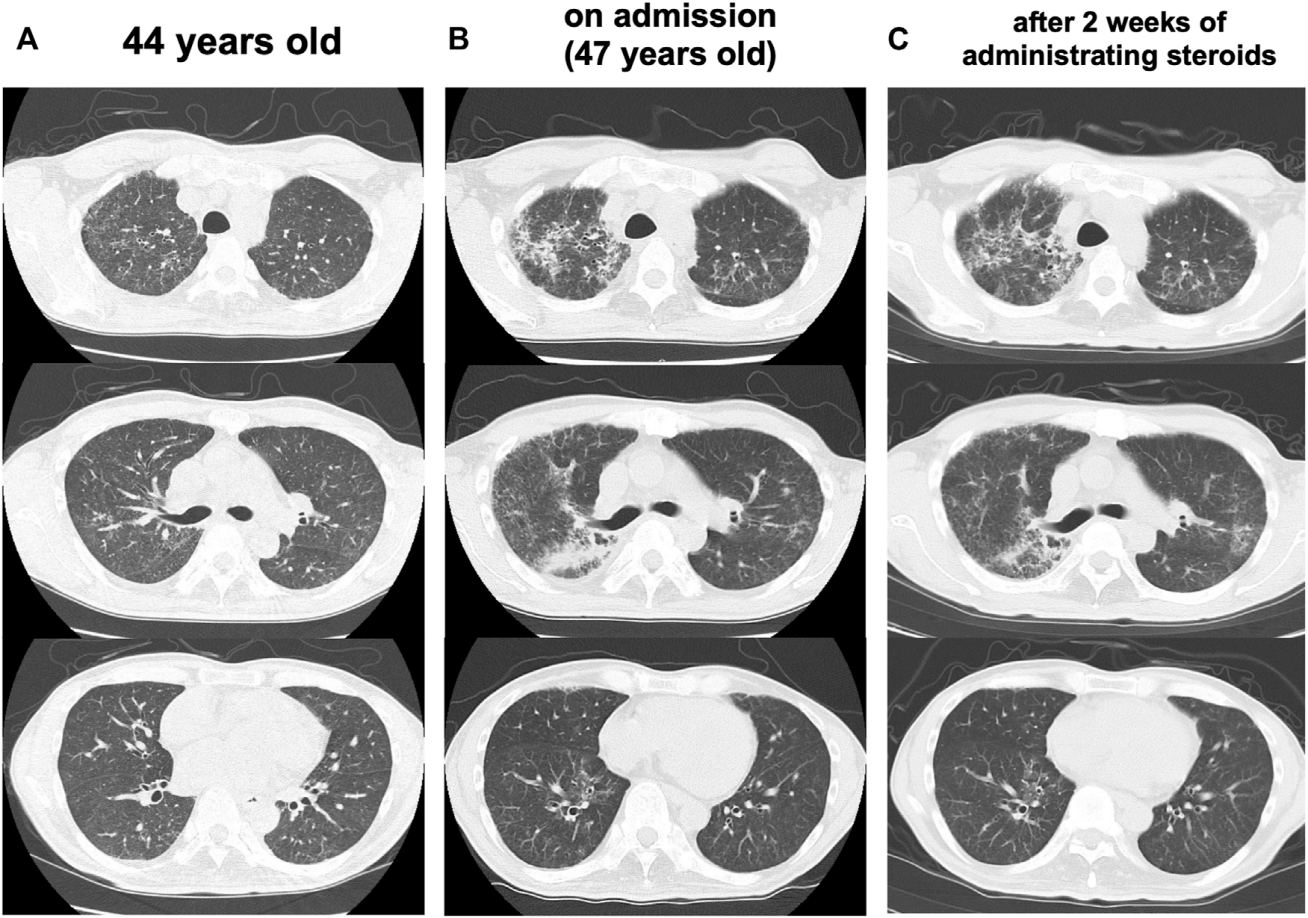
Progress of lung CT scans **(A)** At 44 years old **(B)** On admission (47 years old), the interstitial lung shadows worsened **(C)** 2 weeks after administrating corticosteroids, interstitial shadows were improved.

At the age of 47 years, his shortness of breath and interstitial lung shadows on chest CT worsened compared to those at 44 years of age (Figures 3A,B) immediately after recovery from bacterial pneumonia. His oxygen saturation was 94% in ambient air. Bilateral fine crackles were detected predominantly in the right upper fields on chest auscultation. Pulmonary function tests showed a restrictive ventilatory impairment (vital capacity [VC], 1.56 L; VC % predicted, 45.7%) and a decline in diffusing capacity (diffusing capacity for carbon monoxide % predicted [DLCO % predicted], 42.6%). The ratio of residual volume to total lung capacity was elevated (38.1%), indicating the presence of respiratory muscle weakness. Serum levels of Krebs Von den Lungen-6 (KL-6), surfactant protein A (SP-A), and surfactant protein D (SP-D) were elevated to 1,085 U/mL, 98.3 ng/ml, 505.6 ng/ml, respectively. Bronchoalveolar lavage (BAL) from the right upper lobe (B^3^b) revealed an increased proportion of neutrophils and eosinophils (macrophages, 82.0%; lymphocytes, 7.0%; neutrophils, 6.7%; eosinophils, 4.3%; CD4/CD8 ratio, 0.66) in the BAL fluid. Echocardiography results were within the normal limits. Based on these findings, an exacerbation of interstitial pneumonia was considered, and 40 mg/day of prednisolone was administered. 2 weeks later, his shortness of breath, interstitial shadows on the chest CT scan (Figure 3C), and markers of interstitial pneumonia improved. Serum levels of SP-A and SP-D decreased (62.9 ng/ml and 321.4 ng/ml, respectively), although serum KL-6 unchanged. Pulmonary function tests showed improvements in %VC and %DLCO (49.5 and 54.5%, respectively). However, shortly thereafter, he abruptly developed severe hepatic encephalopathy. Although he temporarily recovered after treatment with amino acid preparation and lactulose, there was no more effective treatment since he was already in a phase of hepatorenal syndrome. He finally developed severe hypoxemia due to worsening of bilateral pneumonia and pulmonary edema, which led to death within a few days. Subsequently, an autopsy was performed.

**FIGURE 4:**
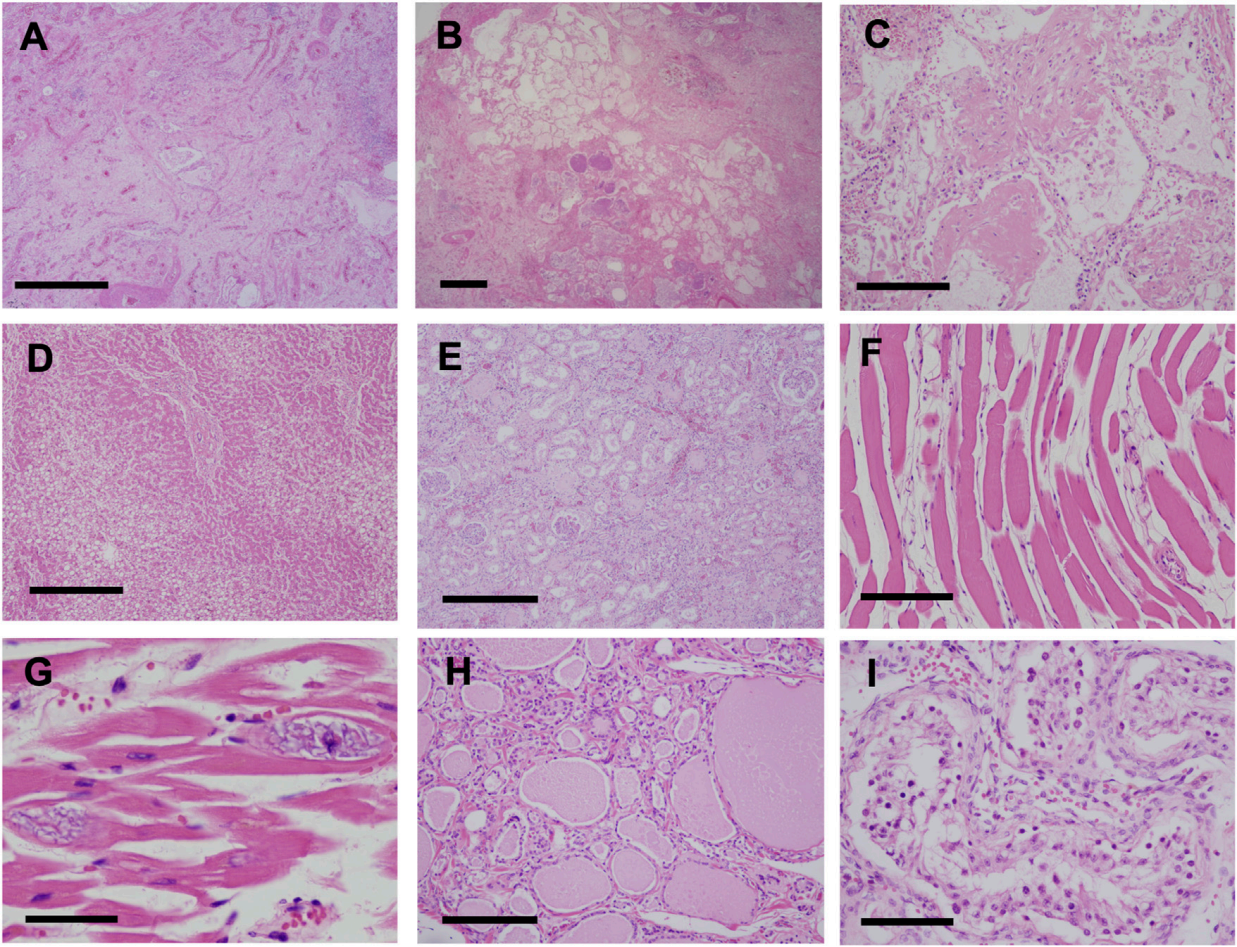
Pathological findings (H&E stain) **(A,B)** Fibrotic changes with unclassifiable pattern predominantly in the right upper lobe of the lung **(A)** Fibrotic-nonspecific interstitial pneumonia (f-NSIP) pattern (scale bar, 1.0 mm) **(B)** Usual interstitial pneumonia (UIP) pattern and bacterial pneumonia with gram-positive cocci (scale bar, 1.0 mm) **(C)** Organizing pneumonia (scale bar, 200 μm) **(D)** Liver tissue showed fibrosis and fatty degeneration (scale bar, 500 μm) **(E)** Interstitial fibrosis, glomerular sclerosis, and tubular atrophy in the kidneys (scale bar, 500 μm). Skeletal muscles (scale bar, 200 μm) **(F)** and the myocardium (scale bar, 100 μm) **(G)** showed atrophy, fatty degeneration, and basophilic degeneration **(H)** Thyroid tissue showed atrophy and interstitial fibrosis (scale bar, 200 μm) **(I)** Testis tissue showed atrophy of the seminiferous tubules, indicating male infertility (scale bar, 100 μm).

Pathological findings in the lungs revealed fibrotic changes with an unclassifiable pattern, predominantly in the right upper lobe (Figures 4A,B). Findings of organizing pneumonia and bacterial pneumonia with gram-positive cocci were also noted (Figures 4B,C). Pulmonary edema was observed in a large part of the lungs, and alveolar hemorrhage was seen in the right middle lobe and left lung. Liver tissue showed fibrosis and steatosis (Figure 4D), but findings indicative of nonalcoholic steatohepatitis were not observed. Kidney tissue showed interstitial fibrosis, glomerular sclerosis, and tubular atrophy (Figure 4E). Skeletal muscles and the myocardium showed atrophy, fatty degeneration, and basophilic degeneration (Figures 4F,G). Pancreatic tissue showed severe atrophy and fatty infiltration with scarce exocrine glands. Thyroid tissue showed atrophy and interstitial fibrosis (Figure 4H). Laryngeal tissue showed atrophy and degeneration in the cricothyroid and thyroarytenoid muscles, which led to vocal cord atrophy. The testis tissue showed atrophy of seminiferous tubules, indicating male infertility (Figure 4I).

## Discussion

The first family affected by POIKTMP was reported in 2006 (Khumalo et al., 2006). In 2013, Mercier et al. identified FAM111B as a gene responsible for this disease (Mercier et al., 2013). FAM111B is located on chromosome 11 (11q12.1) and encodes a protein with a trypsin-like cysteine/serine peptidase domain. Recently, it was reported that the FAM111B mutation was possibly associated with cancer, including pancreatic cancer and lung adenocarcinoma (Mercier et al., 2019; Sanada et al., 2019; Sun et al., 2019). It is inherited in an autosomal dominant manner, and 50% of cases are sporadic. To the best of our knowledge, there have been only 15 families with 34 patients diagnosed with this disease worldwide, and the present case is the first Japanese patient with POIKTMP. In contrast, several patients with poikiloderma might be misdiagnosed with RTS, which is another genetic disorder that causes poikiloderma (Lessem et al., 1980; Otsu et al., 2008; Meier and Schwarz 2012).

The novel missense mutation was detected in the present case. We evaluated it *in silico*, and the results suggested its pathogenicity. However, the cellular functions of FAM111B and the impact of its genetic mutations have not been fully elucidated. Hoffmann et al. recently discovered that FAM111B harbors intrinsic proteolytic activity and that mutations in FAM111B undermine cellular fitness by eliminating inhibitory constraints on its protease activity, blocking DNA and RNA synthesis, and promoting apoptotic cell death (Hoffmann et al., 2020). In their research, they evaluated two mutations among those reported as pathogenic mutations for POIKTMP. A similar analysis of the present mutation could be useful to confirm its pathogenicity.

Furthermore, an important new insight with implications for genotype-phenotype correlation was recently reported. Arowolo et al. suggested that patients with mutations within the putative protease domain of the FAM111B protein may have higher disease burden and poorer prognoses compared to patients with mutations outside the domain (Arowolo et al., 2022). Among the reported causative mutations in FAM111B, all except three are within this domain (Figure 2C), as is the present mutation. That our patient had multiple organ impairment leading to a poor prognosis would seem to support this suggestion.

POIKTMP often causes poikiloderma, muscle weakness, and tendon contracture. Some patients develop pulmonary fibrosis, which has been reported to lead to poor prognosis. In a previous review, only symptomatic therapy was recommended for those with respiratory failure (Arowolo et al., 2022). Only two patients with POIKTMP have been reported to be treated for pulmonary involvement. The first patient was a South African man who experienced dyspnea at 28 years of age (Sanchis-Borja et al., 2018). The percentages of neutrophils and eosinophils were elevated in the BAL fluid. High-dose corticosteroid therapy was ineffective, and respiratory failure progressed rapidly, which led to death 15 months later. Autopsy revealed pulmonary fibrosis with a usual interstitial pneumonia pattern in the lungs (Sanchis-Borja et al., 2018). The second patient was a French man who developed dyspnea at 38 years of age (Mercier et al., 2015; Sanchis-Borja et al., 2018). The levels of eosinophils in BAL fluid were elevated. He died after experiencing several exacerbations and remissions for 2 years. The antifibrotic drug pirfenidone could not be tolerated due to its adverse effects. Pulse steroid therapy and cyclophosphamide were administered; however, these treatments were completely ineffective. To the best of our knowledge, the present case describes the first reported patient with POIKTMP, whose interstitial pneumonia responded to corticosteroid therapy. Worsening of his pulmonary lesions was observed after recovery from bacterial pneumonia, and autopsy revealed the presence of organizing pneumonia with an unclassifiable pattern of interstitial pneumonia; therefore, corticosteroid therapy in the present case might be mainly effective for the lesions of organizing pneumonia among unclassifiable interstitial pneumonia.

Autopsy revealed the presence of atrophy and fatty degeneration in multiple organs, which suggests that the FAM111B mutation systemically impairs organ function through its enhanced proteolytic activity. Of note, the present case showed liver cirrhosis. As far as we know, there has been only 1 patient with POIKTMP who had liver cirrhosis. He had progressive liver impairment and vanishing duct syndrome from infancy, and died due to decompensated liver cirrhosis at 17 years of age (Mercier et al., 2016) (Roversi et al., 2021). The cause of cirrhosis was not described. Our patient had not been infected with hepatitis B or hepatitis C virus, and autopsy findings showed no evidence of nonalcoholic steatohepatitis. Although the pathogenesis of liver cirrhosis in POIKTMP is unclear, fibrosis seems to be one of the key features of this disease.

The present case also had chronic kidney disease with interstitial fibrosis, glomerular sclerosis, and tubular atrophy. There was only one patient with renal failure, which was supposed to be associated with POIKTMP (Meier and Schwarz 2012). The patient was a Dutch woman initially reported as having RTS, although Mercier et al. later pointed out that she had POIKTMP. In addition, testis tissue of the present case showed atrophy of seminiferous tubules that indicated male infertility, which has not been reported as a feature of POIKTMP.

In conclusion, we report the first Japanese case of POIKTMP whose interstitial pneumonia was improved by corticosteroids, suggesting that corticosteroids could be an option for the treatment of interstitial pneumonia associated with this disease.

## Data Availability

The datasets for this article are not publicly available due to concerns regarding participant/patient anonymity. Requests to access the datasets should be directed to the corresponding author.

## Abbreviations

POIKTMP, hereditary fibrosing poikiloderma with tendon contractures, myopathy, and pulmonary fibrosis; CT, computed tomography; PCR, polymerase chain reaction; RTS, Rothmund-Thomson syndrome; VC, vital capacity; DLCO, diffusing capacity for carbon monoxide; KL-6, Krebs Von den Lungen-6; SP-A, surfactant protein A; SP-D, surfactant protein D; BAL, bronchoalveolar lavage; H&E, Hematoxylin and eosin; f-NSIP, fibrotic-nonspecific interstitial pneumonia; UIP, usual interstitial pneumonia.
