# Supplementary material for: Case Report: Hereditary Fibrosing Poikiloderma With Tendon Contractures, Myopathy, and Pulmonary Fibrosis (POIKTMP) Presenting With Liver Cirrhosis and Steroid-Responsive Interstitial Pneumonia
Source: Front Genet. 2022 May 5;13:870192. doi: 10.3389/fgene.2022.870192 (PMC9117717; doi:10.3389/fgene.2022.870192)
Supplement: Supplementary file 1 [file DataSheet1.PDF]

CARE case report guidelines

CARE Checklist of information to include when writing a case report

(cc) BY-NC-ND

Topic	Item	Checklist item description	Reported on Line
Title	1	The diagnosis or intervention of primary focus followed by the words "case report"	Title, page 1
Key Words	2	2 to 5 key words that identify diagnoses or interventions in this case report, including "case report"	Key words, page 1
Abstract (no references)	3a	Introduction: What is unique about this case and what does it add to the scientific literature?	Abstract, page 2
	3b	Main symptoms and/or important clinical findings	Abstract, page 2
	3c	The main diagnoses, therapeutic interventions, and outcomes	Abstract, page 2
	3d	Conclusion—What is the main "take-away" lesson(s) from this case?	Abstract, page 2
Introduction	4	One or two paragraphs summarizing why this case is unique (**may include references**)	Introduction, page3
Patient Information	5a	De-identified patient specific information.	Case description, page3
	5b	Primary concerns and symptoms of the patient.	Case description, page3-4
	5c	Medical, family, and psycho-social history including relevant genetic information	Case description, page3-4
	5d	Relevant past interventions with outcomes	N/A
Clinical Findings	6	Describe significant physical examination (PE) and important clinical findings.	Case description, page3-4
Timeline	7	Historical and current information from this episode of care organized as a timeline	Case description, page3-4
Diagnostic Assessment	8a	Diagnostic testing (such as PE, laboratory testing, imaging, surveys).	Diagnostic assessment, page4
	8b	Diagnostic challenges (such as access to testing, financial, or cultural)	Diagnostic assessment, page4
	8c	Diagnosis (including other diagnoses considered)	Diagnostic assessment, page4
	8d	Prognosis (such as staging in oncology) where applicable	N/A
Therapeutic Intervention	9a	Types of therapeutic intervention (such as pharmacologic, surgical, preventive, self-care)	Diagnostic assessment, page4-5
	9b	Administration of therapeutic intervention (such as dosage, strength, duration)	Diagnostic assessment, page5
	9c	Changes in therapeutic intervention (with rationale)	Diagnostic assessment, page4
Follow-up and Outcomes	10a	Clinician and patient-assessed outcomes (if available)	Diagnostic assessment, page4-5
	10b	Important follow-up diagnostic and other test results	Diagnostic assessment, page4-5
	10c	Intervention adherence and tolerability (How was this assessed?)	Diagnostic assessment, page5
	10d	Adverse and unanticipated events	Diagnostic assessment, page5
Discussion	11a	A scientific discussion of the strengths AND limitations associated with this case report	Discussion, page 5-7
	11b	Discussion of the relevant medical literature **with references**.	Discussion, page 5-7
	11c	The scientific rationale for any conclusions (including assessment of possible causes)	Discussion, page 5-7
	11d	The primary "take-away" lessons of this case report (without references) in a one paragraph conclusion	Conclusion, page 7
Patient Perspective	12	The patient should share their perspective in one to two paragraphs on the treatment(s) they received	Patient Perspective, page 8
Informed Consent	13	Did the patient give informed consent? Please provide if requested	Yes ☑ No ☐
